# The reach and benefits of a digital intervention to improve physical activity in people with a musculoskeletal condition delivered during the COVID-19 pandemic in the UK

**DOI:** 10.1177/17579139221085098

**Published:** 2022-04-03

**Authors:** J Webb, R Horlock, A Ahlquist, A Hall, K Brisby, S Hills, D Stewart

**Affiliations:** School of Social Sciences and Professions, London Metropolitan University, 166-220 Holloway Rd, London N7 8DB, UK; Versus Arthritis, London, UK; Versus Arthritis, London, UK; Versus Arthritis, London, UK; Versus Arthritis, London, UK; Guildhall School of Business and Law, London Metropolitan University, London, UK; School of Social Sciences and Professions, London Metropolitan University, London, UK

**Keywords:** COVID-19, physical activity, digital intervention, musculoskeletal condition, COM-B, behaviour change wheel

## Abstract

**Aim:**

To evaluate a digital intervention to improve physical activity in people in the UK with a musculoskeletal condition delivered during movement restrictions brought about because of the COVID-19 pandemic.

**Method:**

Service evaluation data collected from 26,041 participants over 5 months was assessed against national datasets to understand the reach and representativeness of the digital physical activity intervention. Measures to restrict the movement and interaction of people were in place during these 5 months. Cross-sectional data from 2752 participants across different stages of the 12-week programme assessed levels of physical activity and the components of behaviour as defined by the COM-B model (Capability, Opportunity, Motivation = Behaviour). Regression analysis investigated the relationship between programme stage and physical activity and the components of behaviour.

**Results:**

In comparison to the UK population of people with a musculoskeletal condition, the intervention participants were over-represented by females, White, and inactive people. A cross-sectional analysis suggested that the number of participants regularly active increased by programme stage. Scores for the behavioural components of automatic and reflective motivation, physical and psychological capability, and physical opportunity were also improved by programme stage.

**Conclusion:**

The service evaluation suggests that the digital intervention, designed to improve physical activity in people with a musculoskeletal condition, could be beneficial during measures to restrict movement to slow the spread of infectious disease in those who are already motivated to become or stay active.

## Introduction

The COVID-19 pandemic resulted in restrictions across many countries limiting the movement and interaction of people, including stay at home orders that altered how people undertook physical activity. On 20 March 2020, the Prime Minister of the UK announced the first of three national lockdowns to slow the spread of SARS-CoV-2. The measures to restrict movement and interaction of people were entered into law on 26 March 2020. These measures began to ease from May 2020 but were again tightened as a second wave of infections hit; the UK Government announced a second lockdown in November 2020. After an initial lifting of some restrictions in December 2020, a third lockdown was announced in January 2021. Restrictions began to be lifted in March 2021.^1^

Social distancing and restrictions on movement impacted physical activity levels across populations. The lockdown restrictions closed leisure facilities and limited people to one outdoor activity a day with members of their own household meaning that many of the ways that people used to exercise were no longer available. Sport England report that adult activity levels reduced during the COVID-19 pandemic owing to diminishing opportunities due to restriction on permitted activity as well as diminished motivation and sense of capability.^2^ As a result, the importance of digital platforms promoting physical activity increased, offering an alternative solution to becoming or staying active.^3^

Even prior to the COVID-19 pandemic, Public Health England (now the Office for Health Improvement and Disparities) advocated the use of digital interventions to influence healthy lifestyle behaviours.^4^ Griffiths et al.^5^ highlight the paucity of high-quality evidence evaluating the impact of digital interventions on physical activity in people with arthritis, who experience long-term challenges to staying active. To address this gap in the literature, this study evaluates Let’s Move with Leon, developed by UK charity Versus Arthritis and designed to improve physical activity in people with a musculoskeletal condition.

### Development of Let’s Move with Leon

Physical activity has many benefits for people with a musculoskeletal condition, such as pain reduction, improved physical function and mental wellbeing, and protection against other long-term conditions such as heart disease and diabetes.^6^ However, even before the impact of the COVID-19 pandemic, many people with a musculoskeletal condition in the UK were not active to the required levels, with almost a third classified as completely inactive.^7^

Interventions with a theoretical grounding stand the best chance of success.^5^ Let’s Move with Leon is an online intervention developed using the Behaviour Change Wheel (BCW).^8^ The BCW has a behavioural model at its centre, the COM-B model (Capability, Opportunity, Motivation = Behaviour), which suggests that behaviour is made up of six components: psychological and physical capability, social and physical opportunity, and reflective and automatic motivation.^8^ The BCW incorporates three stages to designing behaviour change interventions: (1) understanding the target behaviour, (2) designing the intervention, and (3) intervention delivery.^8^ The intervention development process began in September 2019, prior to the then unforeseen COVID-19 pandemic. Once the pandemic hit and the resulting movement restrictions were enforced, the pace and intensity of intervention development increased so to launch the digital intervention as quickly as possible. Let’s Move with Leon was launched on 16 September 2020.

To understand the target behaviour, informal face-to-face discussions took place in July 2019 with 100 people with a musculoskeletal condition through support groups from across the UK and from a Versus Arthritis Volunteering conference held in Wales. In addition, the Versus Arthritis Online Community was reviewed for mentions of exercise or physical activity^9^ and 815 people with a musculoskeletal condition were surveyed^10^ to capture data on the barriers and facilitators to being activity.

An ad hoc review of the literature was undertaken to understand the capability, opportunity, and motivational barriers and facilitators to physical activity for people with a musculoskeletal condition.

To further understand the target behaviour and possible intervention delivery options, conversations took place in late 2019 with 25 healthcare professionals involved in the design, development, and delivery of services and activities for people with musculoskeletal conditions. This was followed by three intervention development workshops held between November 2019 and February 2020 with members of the Versus Arthritis Digital, Partnerships, and Health Information teams, a person with arthritis, with representation from Sport England, an arms-length body of government responsible for getting more people active.

The intervention development workshops were facilitated by R.H. and J.W., working through the BCW stages to design, develop, and plan delivery of the intervention. Finally, an advisory group of 41 stakeholders including healthcare professionals, physical activity professionals, academics, and patient representatives was established to check and challenge the intervention development process. Over the course of intervention development, the group met on three occasions.

### Intervention components

Let’s Move with Leon is comprised of 12 pre-recorded YouTube exercise sessions, each lasting around 30 min in length, details of which are sent weekly over email, coupled with a 35-page Activity Tracker, which can be printed or completed digitally. In addition, intervention users have access to an online Activity Hub which provides introductory videos, videos on how to get started with the programme, and videos on how to get up and down from the floor safely. Users can access a frequently asked questions section, an online community and information about the benefits of physical activity. The use of intervention functions, behaviour change techniques, and policy categories as outlined in the BCW^11^ are presented in Supplementary Tables 1 and 2.

### The aim of this article

This article aims to assess the reach and the representativeness of users of Let’s Move with Leon during the UK COVID-19 restrictions. Furthermore, this article aims to examine differences in physical activity and the capability, opportunity, and motivation of its participants to be physically active at different stages of the programme.

## Method

### Study design

This is a service evaluation defined by the National Research Ethics Service^12^ as an evaluation to understand how well a service is achieving its intended aims and benefitting service users with the results informing future decision-making. This evaluation uses secondary data collected by Versus Arthritis as part of service delivery. Anonymised data was made available to researchers at London Metropolitan University for the purposes of this service evaluation.

### Service evaluation data

Service evaluation data was collected by Versus Arthritis from 26,041 users who signed up to Let’s Move with Leon between 16 September 2020 and 25 February 2021. Data was collected at sign-up on gender, year of birth, ethnicity, musculoskeletal condition, levels of physical activity, and how they heard about the programme. In addition, participants answered questions regarding their self-efficacy for individual development, their confidence in maintaining lifestyle change, the impact of their condition on daily life, their ability to lessen this impact, and their beliefs on the benefits of lifestyle changes in relation to their condition and its management. The measures used are presented in Supplementary Table 3.

Versus Arthritis collected cross-sectional data from 2752 participants across different stages of the programme in February 2021. The cross-sectional survey assessed levels of physical activity and the components of behaviour as defined by the COM-B model being physical and psychological capability, social and physical opportunity, and reflective and automatic motivation.^8,13^ The cross-sectional data was matched to the programme sign-up data where available. The cross-sectional survey is available in Supplementary File 1.

### Data analysis

To assess intervention reach and representativeness, participant characteristics were compared to national datasets where available. The cross-sectional survey data was assessed for the relationship between programme stage, physical activity, and COM-B component using regression analysis. An adjusted model, using the match cross-sectional and participant sign-up data, controlled for age, gender, ethnicity and sign-up scores for quality of life, the ability to achieve goals, impact of condition, ability to self-manage, perceived control over condition, understanding of healthy lifestyles, and the ability to maintain physical activity in times of stress. All variables were entered into the model; complete matched data was only available for 495 of the cross-sectional participants.

## Results

### Reach and representativeness

It is estimated that in 2017 18.8 million people in the UK had a musculoskeletal condition. Between 16 September 2020 and 25 February 2021, 26,041 participants signed up to Let’s Move with Leon, 0.14% of the eligible population. Most Let’s Move with Leon participants (59.99%) heard about the programme through a Versus Arthritis communication channel (website, publication, email, or social media) and 36.34% heard about the programme through adverts communicated through Facebook. A full breakdown of how participants came to hear of Let’s Move with Leon is presented in Supplementary Table 4. The reach of the Let’s Move with Leon promotional activity is not known; however, it is reported that Versus Arthritis had 2.2 million interactions with people with a musculoskeletal condition in 2019.^14^ Therefore, it is possible to calculate a crude reach figure of 1.18%. The representativeness of the Let’s Move with Leon users is presented in Table 1.

**Table 1:** Characteristics of the Let’s Move with Leon users compared to UK population estimates where available

Characteristic	Let’s Move (*n*)	Let’s Move (%)^a^	UK population estimates (%)	Difference (%)
Gender^b^				
Male	2300	8.83	44.15	–35.32
Female	23,700	91.01	55.85	35.16
Other	41	0.16		
Age range^b^				
<35	308	1.20	16.32	–15.12
35–64	13,812	53.82	49.62	4.21
65+	11,541	44.97	34.06	10.91
Data not provided or spoiled	380			
Ethnicity^c^				
White	24,715	97.47	91.68	5.79
All other ethnic groups combined	642	2.53	8.32	–5.79
Data not provided	684			
Condition^b^				
Inflammatory arthritis or autoimmune disease	8892	37.41	–	–
Osteoarthritis	13,052	54.92	–	–
Chronic joint pain	17,471	73.51	–	–
Osteoporosis	2400	10.10	–	–
Other	321	13.51	–	–
Multiple conditions (included in the figures above)	13,821	58.15	–	–
Data not provided	2274			
Physical Activity status^d^				
Regularly active^e^	4970	24.26	29.00	–4.74
Fairly active	2888	14.10	27.00	–12.90
Inactive	12,627	61.64	44.00	17.64
Data not provided or spoiled	5556			
Quality of life				
Good or very good	8961	39.08	–	–
Neither good nor poor	7800	34.02	–	–
Poor or very poor	6169	26.90	–	–
Data not provided	3111			
Impact of musculoskeletal condition				
None at all	224	0.96	–	–
Mild or very mild	3192	13.66	–	–
Moderate	11,288	48.28	–	–
Severe or very severe	8675	37.11	–	–
Data not provided	2662			

a Percentages are calculated from the data available excluding missing and spoiled data from the total.

b Estimates taken from Versus Arthritis.^7^

c Based on age-standardised population estimates from the Office of National Statistics.^15^

d Estimates taken from a 2019 Versus Arthritis survey.^10^

e Regular physical activity is defined as 150 min of moderate intensity activity each week.^16^

The Let’s Move with Leon users were over representative of females, White people, and older people with very little representation from those under the age of 35, just 1.20% of participants. The mean age of participants was 65 years with 72% of participants aged between 55 and 75 years. Let’s Move with Leon users were more likely to be inactive than the population of people with a musculoskeletal condition (62% vs 44%) at programme initiation, which may be due to the measures to limit movement to slow the spread of SARS-CoV-2; it is noted that the measures used to assess physical activity differ and this may have impacted upon this result.

Table 2 reports on participants’ self-efficacy for individual development, confidence in maintaining lifestyle change, knowledge and perceived benefits of lifestyle changes in relation to their condition, and its management and their ability to lessen the impact of their condition.

**Table 2:** Self-efficacy, knowledge and confidence of Let’s Move with Leon users at programme initiation to make lifestyle changes

Question	Agree	Neither agree nor disagree	Disagree	Data not provided or spoiled
	*n* (%)^a^	*n* (%)^a^	*n* (%)^a^	*n*
I can achieve most goals I set myself	4052 (19.36%)	7973 (38.09%)	8906 (42.55%)	5110
Making lifestyle changes could improve the management of my condition	18,871 (87.27%)	2488 (11.51%)	264 (1.22%)	4418
I often feel like I cannot do anything myself to lessen the impact of my condition	7707 (35.47%)	6752 (31.07%)	7270 (33.46%)	4312
I understand what contributes to a healthy lifestyle	19,979 (87.70%)	2389 (10.49%)	412 (1.81%)	3261
I am confident I can maintain lifestyle changes even during times of stress	9048 (41.69%)	6862 (31.61%)	5795 (26.70%)	4336

a Percentages are calculated from the data available excluding missing and spoiled data from the total.

Most Let’s Move with Leon users at programme initiation had a good understanding of how to make lifestyle changes to support condition management (87.27%) with an understanding of what constitutes a healthy lifestyle (87.70%) but were less likely to be confident in their ability to be able to maintain lifestyle changes in times of stress (41.69%) and achieve the goals that they set themselves (19.36%).

### Analysis of the cross-sectional data

**Figure 1:**
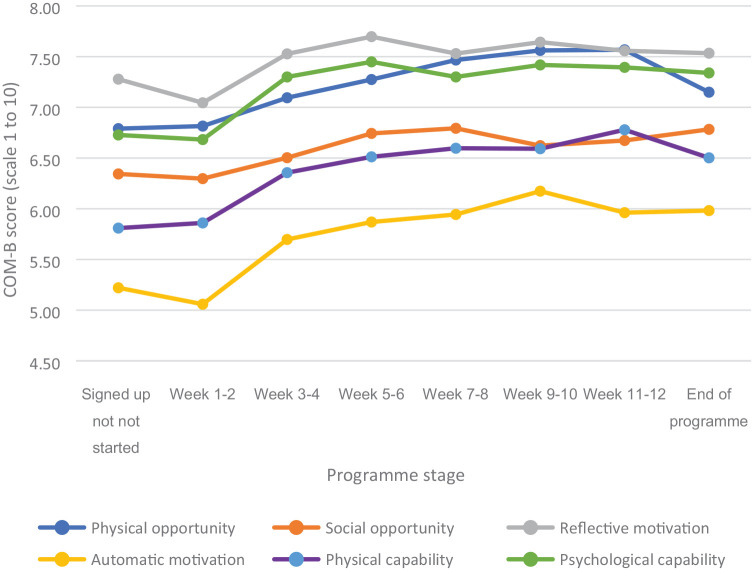
Mean COM-B score (out of 10) by programme stage from a cross section of Let’s Move with Leon participants (n = 2752)

The characteristics of participants in the cross-sectional survey were broadly similar to the full Let’s Move with Leon user population in terms of age, gender, and ethnicity. The cross-sectional survey participant characteristics are available in Supplementary Tables 5 through 7. Table 3 and Figure 1 present an analysis of the cross-sectional participant scores for the behavioural components of physical capability, psychological capability, social and physical opportunity, and reflective and automatic motivation.

**Table 3:** Mean COM-B score (out of 10) by programme stage from a cross section of Let’s Move with Leon users (*n* = 2752)

Programme stage (*n*)	Physical opportunity	Social opportunity	Reflective motivation	Automatic motivation	Physical capability	Psychological capability
	Mean (SD)	Mean (SD)	Mean (SD)	Mean (SD)	Mean (SD)	Mean (SD)
Signed up but not started (*n* = 367)	6.79 (2.34)	6.34 (2.67)	7.28 (2.32)	5.22 (2.36)	5.81 (2.40)	6.73 (2.31)
Week 1–2 (579)	6.82 (2.42)	6.30 (2.61)	7.04 (2.26)	5.06 (2.38)	5.86 (2.49)	6.68 (2.38)
Week 3–4 (*n* = 624)	7.09 (2.15)	6.50 (2.48)	7.53 (1.98)	5.70 (2.30)	6.36 (2.33)	7.30 (2.05)
Week 5–6 (*n* = 459)	7.27 (2.20)	6.74 (2.57)	7.70 (1.90)	5.87 (2.28)	6.51 (2.40)	7.45 (2.05)
Week 7–8 (*n* = 300)	7.47 (2.09)	6.79 (2.56)	7.53 (2.10)	5.94 (2.36)	6.60 (2.36)	7.30 (2.11)
Week 9–10 (*n* = 98)	7.56 (2.25)	6.62 (2.71)	7.64 (2.08)	6.17 (2.42)	6.59 (2.50)	7.42 (2.41)
Week 11–12 (*n* = 104)	7.57 (2.03)	6.67 (2.52)	7.56 (2.26)	5.96 (2.50)	6.78 (2.24)	7.39 (2.12)
End of programme (221)	7.15 (2.33)	6.78 (2.63)	7.53 (2.18)	5.98 (2.50)	6.50 (2.39)	7.34 (2.32)

SD: standard deviation.

An unadjusted regression analysis suggested small but significant improvements across all components of behaviour as programme stage increased. These significant findings remained unchanged in the adjusted model with the exception of social opportunity which did not see a significant increase. The regression analysis indicated that physical activity increased as programme stage increased in both the unadjusted and adjusted models (unadj odds ratio (OR): 1.164, 95% confidence interval (CI) (1.119 to 1.210), *p* < .001; adj OR: 1.161, 95% CI (1.052 to 1.281), *p* < .01) (Table 4).

**Table 4:** Regression analysis of the relationship between programme stage and physical activity and the COM-B components

	Unadjusted (*n* = 2751)		Adjusted (*n* = 495)^a^	
	OR	95% CI	OR	95% CI
Regular physical activity^b^	1.164***	1.119–1.210	1.161**	1.052–1.281
Physical opportunity	0.098***	0.056–0.141	0.134**	0.034–0.234
Social opportunity	0.080***	0.032–0.129	0.067	-0.041–0.176
Reflective motivation	0.069***	0.029–0.109	0.147**	0.053–0.241
Automatic motivation	0.152***	0.108–0.196	0.198***	0.094–0.302
Physical capability	0.133***	0.088–0.178	0.174***	0.067–0.281
Psychological capability	0.113***	0.071–0.154	0.155**	0.058–0.252

OR: odd ratio; CI: confidence interval. N.B: Programme stage units were coded 1 to 8 with 1 being signed up but not started; 2, in week 1 or 2 of the programme; 3, in weeks 3 or 4 of the programme; 4, in weeks 5 or 6 of the programme; 5, in weeks 7 or 8 of the programme; 6, in weeks 9 or 10 of the programme; 7, in weeks 11 or 12 of the programme; and 8, at the end of the programme.

a Adjusted model controlled for age, gender, ethnicity, and programme sign-up scores for quality of life, the ability to achieve goals, impact of condition, ability to self-manage, perceived control over condition, understanding of healthy lifestyles and the ability to maintain physical activity in times of stress.

b Regular physical activity is defined as 150 min of moderate intensity activity each week.^16^ **p < .01; ***p < .001.

## Discussion

This article set out to evaluate a digital physical activity intervention delivered during the COVID-19 pandemic in the UK. The digital intervention, Let’s Move with Leon, was assessed for its reach, the representativeness of its participants to the UK population of people with a musculoskeletal condition, and its potential benefits for physical activity and the components of this behaviour.

An evidence-based approach was taken to develop the Let’s Move with Leon digital intervention, first understanding the behaviour of physical activity in people with a musculoskeletal condition, before using the BCW to design the intervention. The development of Let’s Move with Leon directly involved 100 people with a musculoskeletal condition and 66 professionals. A large number of people with a musculoskeletal condition in the UK signed up to Let’s Move with Leon during the COVID-19 pandemic between the months of September 2020 and February 2021 (*n* = 26,041). However, there is a significant amount of missing service evaluation data at programme sign-up, up to 21.34% depending on the question. From the data available, it is suggested that users of Let’s Move with Leon are most likely to be aged 55–75 years (72% of participants), female (91%), White (97%), with chronic joint pain (84%), with their condition having a moderate to severe impact on daily life (85%), and with a moderate to good quality of life (73%). At programme initiation users are most likely to be inactive (61%), with a good understanding of what contributes to a healthy lifestyle (88%) and with the knowledge that lifestyle changes could improve their condition (87%).

While the profile of those who signed up to Let’s Move with Leon is not representative of the broader population of people with a musculoskeletal condition in the UK, this is not unexpected as behaviour change interventions are not one size fits all.^17^ That said, action should be taken to investigate the underrepresentation of users from ethnic groups other than White, males, and younger people. The participants may represent those that are more likely to engage with a digital intervention during a period where restrictions to movement are in place, but it is probable that they also represent those that are more likely to engage with Versus Arthritis, the charity that developed Let’s Move with Leon. Advertising through Facebook seemed to be effective at engaging participants in this intervention with 36.34% of participants coming through this route.

The behavioural components of reflective and automatic motivation, physical and psychological capability, and physical opportunity increased with programme stage. Interventions which encourage engagement stand the best chance of success;^5^ however, increasing social opportunities in an online setting is challenging. The Let’s Move with Leon programme directs users to a Facebook group which has 7476 members, suggesting that only 28.71% of the Let’s Move with Leon participants made use of this group; this may explain why the scores for social opportunity did not increase by programme stage. It is noted that the reflective motivation scores reported from the cross-sectional participants yet to start the programme were high (7.28/10), suggesting that people drawn to this programme were already motivated to make a change despite the lockdown measures and social movement restrictions.

The chance of participants being regularly physically active increased by programme stage. This suggests that Let’s Move with Leon improved physical activity in participants during a time of restricted social movement resulting from the COVID-19 pandemic. However, caution is advised in the interpretation of cross-sectional data as this only shows associations and group differences, not causation.

The data presented in this article was collected during the COVID-19 pandemic, including three UK national stay at home orders with varying degrees of movement restrictions in between. This is a unique situation, with little evidence against which comparisons can be drawn. It has been suggested that engagement with online physical activity programmes increased during lockdown with numbers decreasing afterwards.^18^ It may be that such digital programmes only reach particular population groups, for example, an over-representation of female users has also been reported in other studies.^3,18^ The findings reported in this article should be considered in future digital programme evaluations to enhance understanding of the reach and impact of similar programmes; this is in the national interest as highlighted by the UK Parliament Committee to explore the impact of digital technology on physical activity.^19^

The available data would suggest that a digital intervention, such as Let’s Move with Leon, designed to improve physical activity in people with a musculoskeletal condition, could be impactful during measures to restrict movement to slow the spread of infectious diseases in those who are already motivated to become or stay active. Now that measures to limit movement in the UK have eased, intervention analysis should continue to identify those currently engaging (and those not engaging) with the programme, its use and its impact; a randomised control trial and process evaluation is currently underway to achieve this aim.^20^

## Supplemental Material

sj-docx-1-rsh-10.1177_17579139221085098 – Supplemental material for The reach and benefits of a digital intervention to improve physical activity in people with a musculoskeletal condition delivered during the COVID-19 pandemic in the UK Click here for additional data file. Supplemental material, sj-docx-1-rsh-10.1177_17579139221085098 for The reach and benefits of a digital intervention to improve physical activity in people with a musculoskeletal condition delivered during the COVID-19 pandemic in the UK by J Webb, R Horlock, A Ahlquist, A Hall, K Brisby, S Hills and D Stewart in Perspectives in Public Health

## References

[1] Timeline of UK government coronavirus lockdowns. The Institute for Government. Available online at: https://www.instituteforgovernment.org.uk/charts/uk-government-coronavirus-lockdowns (2021, last accessed 3 November 2021).

[2] Coronavirus. Sport England. Available online at: https://www.sportengland.org/know-your-audience/demographic-knowledge/coronavirus (last accessed 4 November 2021).

[3] Parker K Uddin R Ridgers ND et al. The use of digital platforms for adults’ and adolescents’ physical activity during the COVID-19 pandemic (our life at home): survey study. J Med Internet Res 2021;23:e23389. 10.2196/23389 PMC7857525 33481759

[4] Digital-first public health: Public Health England’s digital strategy. GOV.UK. Available online at: https://www.gov.uk/government/publications/digital-first-public-health/digital-first-public-health-public-health-englands-digital-strategy (last accessed 20 April 2021).

[5] Griffiths AJ White CM Thain PK et al. The effect of interactive digital interventions on physical activity in people with inflammatory arthritis: a systematic review. Rheumatol Int 2018;38:1623–34. 10.1007/s00296-018-4010-8 PMC6105152 29556750

[6] Benjamin E Anna G Tim M. Providing physical activity interventions for people with musculoskeletal conditions. Arthritis UK. Available online at: https://www.versusarthritis.org/policy/policy-reports/providing-physical-activity/

[7] State of Musculoskeletal Health 2019. Versus Arthritis. Available online at: https://www.versusarthritis.org/about-arthritis/data-and-statistics/state-of-musculoskeletal-health-2019/ (last accessed 22 March 2021).

[8] Michie S van Stralen MM West R. The behaviour change wheel: a new method for characterising and designing behaviour change interventions. Implement Sci 2011;6:42. 21513547 10.1186/1748-5908-6-42 PMC3096582

[9] Versus Arthritis Online Community. Versus Arthritis. Available online at: https://community.versusarthritis.org/ (last accessed 10 June 2021).

[10] Helping more people with arthritis to get active. Versus Arthritis. Available online at: https://www.versusarthritis.org/news/2020/august/helping-more-people-with-arthritis-to-get-active/ (last accessed 12 November 2021).

[11] Michie PS Atkins DL West PR. The behaviour change wheel: A guide to designing interventions. London: Silverback Publishing; 2014.

[12] Is my study research? Available online at: http://www.hra-decisiontools.org.uk/research/ (last accessed 27 April 2021).

[13] Keyworth C Epton T Goldthorpe J et al. Acceptability, reliability, and validity of a brief measure of capabilities, opportunities, and motivations (‘COM-B’). Br J Health Psychol 2020;25:474–501. 32314500 10.1111/bjhp.12417

[14] Annual report 2019-20. Available online at: https://www.versusarthritis.org/media/23355/annual-report-2019-2020.pdf (last accessed 12 November 2021).

[15] Age groups. Available online at: https://www.ethnicity-facts-figures.service.gov.uk/uk-population-by-ethnicity/demographics/age-groups/latest (last accessed 12 November 2021).

[16] Everybody active, every day: framework for physical activity. GOV.UK. Available online at: https://www.gov.uk/government/publications/everybody-active-every-day-a-framework-to-embed-physical-activity-into-daily-life (last accessed 16 February 2021).

[17] Kreuter MW Strecher VJ Glassman B. One size does not fit all: the case for tailoring print materials. Ann Behav Med 1999;21:276. 10721433 10.1007/BF02895958

[18] Mutz M Müller J Reimers AK. Use of digital media for home-based sports activities during the COVID-19 pandemic: results from the German SPOVID survey. Int J Environ Res Public Health 2021;18:4409. 33919180 10.3390/ijerph18094409 PMC8122274

[19] Committee to explore the impact of digital technology on physical activity – Committees – UK Parliament. Available online at: https://committees.parliament.uk/committee/460/covid19-committee/news/136714/committee-to-explore-the-impact-of-digital-technology-on-physical-activity/ (last accessed 11 June 2021).

[20] ISRCTN. ISRCTN14174108: an evaluation of the effects of a digital web-based intervention on the physical activity of people with a musculoskeletal condition. Available online at: https://www.isrctn.com/ISRCTN14174108

