# Supplementary material for: The reach and benefits of a digital intervention to improve physical activity in people with a musculoskeletal condition delivered during the COVID-19 pandemic in the UK
Source: Perspect Public Health. 2022 Apr 3;143(2):97–104. doi: 10.1177/17579139221085098 (PMC10067684; doi:10.1177/17579139221085098)
Supplement: sj-docx-1-rsh-10.1177_17579139221085098 – Supplemental material for The reach and benefits of a digital intervention to improve physical activity in people with a musculoskeletal condition delivered during the COVID-19 pandemic in the UK [file sj-docx-1-rsh-10.1177_17579139221085098.docx]

Supplementary Table 1: Content of the Let’s Move with Leon emails and exercise sessions

| **Component** | **Brief description** | **Intervention function** | **Policy category** | **BCT and BCTTv1 code^a^** |
| --- | --- | --- | --- | --- |
| Sign-up webpage. | Brief description of the programme. Highlights that there is no cost; 30 minute sessions per week; no special equipment needed. Leon is identified as an 'exercise expert'. | Education; Enablement. | Communications and marketing; Service provision. | 9.1 Credible source. |
| Email: On your marks, get set… | Brief description of the programme, with signposting to a video on how 'get started' and a second video on how to get down and off the floor, the tracker booklet, activity hub, online community, and facebook group. | Education; Enablement. | Communications and marketing; Service provision. | 3.1 Social support; 4.1 Instructions on how to perform the behaviour; 9.1 Credible source. |
| Video - Getting started (3 minutes). | Tips on how to stay safe during exercise and what you need to get started with signposting to activity tracker and social media. | Education; Enablement; Training. | Communications and marketing; Service provision. | 5.1 Information about health consequences; 6.1 Demonstration of the behaviour; 9.1 Credible source; 12.1 Restructuring the environment |
| Video - Getting down and off the floor (3 minutes). | Video on how to get down and up from the floor. | Education; Training. | Communications and marketing; Service provision. | 4.1 Instructions on how to perform the behaviour; 6.1 Demonstration of the behaviour; 9.1 Credible source. |
| Email: Session 1 - Mobility. | Description of the session with signposting to the exercise video, the tracker booklet, activity hub, online community, and facebook group. | Education; Enablement. | Communications and marketing; Service provision. | 3.1 Social support; 4.1 Instructions on how to perform the behaviour; 6.1 Demonstration of the behaviour; 9.1 Credible source. |
| Video - Exercise session 1 - Mobility (32 minutes). | Exercise session on 'Mobility' with signposting to activity tracker and social media. | Education; Enablement; Training; Modelling. | Communications and marketing; Service provision. | 4.1 Instructions on how to perform the behaviour; 5.1 Information about health consequences; 6.1 Demonstration of the behaviour; 8.2 Behaviour substitution; 8.6 Generalisation of target behaviour; 8.7 Graded tasks; 9.1 Credible source; 12.1 Restructuring the environment; 13.2 Framing/reframing. |
| Email: Session 2 - Movement for life. | Description of the session with signposting to the exercise video, the tracker booklet, activity hub, online community, and facebook group. The email asks participants to go back and complete session 1 if they have not done so as the sessions are gradual. The email also asks participants, following completion of the exercise session, to repeat it again and to record themselves completing a functional movement. | Education; Enablement. | Communications and marketing; Service provision. | 3.1 Social support; 4.1 Instructions on how to perform the behaviour; 8.6 Generalisation of the behaviour; 9.1 Credible source. |
| Video - Exercise session 2 - Movement for life (31 minutes). | Exercise session on 'Movements for life' with signposting to activity tracker and social media. | Education; Enablement; Training; Modelling. | Communications and marketing; Service provision. | 4.1 Instructions on how to perform the behaviour; 5.2 Salience of consequences; 6.1 Demonstration of the behaviour; 8.6 Generalisation of target behaviour; 8.7 Graded tasks; 9.1 Credible source; 13.2 Framing/reframing. |
| Email: Session 3 - Adaptions and exercise tailoring. | Description of the session with signposting to the exercise video, the tracker booklet, activity hub, online community, and facebook group. The email asks participants to go back and complete session 2 if they have not done so and the sessions are gradual. The email also asks participants, following completion of the exercise session, to repeat write down their strengths and weaknesses and complete a 6-minute walking test. | Education; Enablement. | Communications and marketing; Service provision. | 2.3 Self-monitoring of behaviour; 3.1 Social support; 4.1 Instructions on how to perform the behaviour; 5.1 Information about health consequences; 8.7 Graded tasks; 9.1 Credible source. |
| Video - Exercise session 3 -Adaptions and exercise tailoring (31 minutes). | Exercise session on 'Adaptions and exercise tailoring' with signposting to activity tracker and social media. | Education; Enablement; Training; Modelling. | Communications and marketing; Service provision | 2.3 Self-monitoring of behaviour; 4.1 Instructions on how to perform the behaviour; 5.1 Information about health consequences; 5.2 Salience of consequences; 6.1 Demonstration of the behaviour; 8.7 Graded tasks; 9.1 Credible source; 13.2 Framing/reframing. |
| Email: Session 4 - Lower body strength. | Description of the session with signposting to the exercise video, the tracker booklet, activity hub, online community, and facebook group. The email informs participants that as muscles get stronger, pain levels may fall. The email asks participants to go back and complete session 3 if they have not done so and the sessions are gradual. The email also asks participants, following completion of the exercise session, to repeat session and complete a sit-stand test with instructions. | Education; Enablement. | Communications and marketing; Service provision. | 2.3 Self-monitoring of behaviour; 3.1 Social support; 4.1 Instructions on how to perform the behaviour; 5.1 Information about health consequences; 8.7 Graded tasks; 9.1 Credible source. |
| Video - Exercise session 4 - Lower body strength (32 minutes). | Exercise session on 'Lower body strength' with signposting to activity tracker and social media. | Education; Enablement; Training; Modelling. | Communications and marketing; Service provision. | 2.3 Self-monitoring of behaviour; 4.1 Instructions on how to perform the behaviour; 5.1 Information about health consequences; 5.2 Salience of consequences; 6.1 Demonstration of the behaviour; 8.7 Graded tasks; 9.1 Credible source; 13.2 Framing/reframing. |
| Email: Session 5 - Upper body strength. | Description of the session with signposting to the exercise video, the tracker booklet, activity hub, online community, and facebook group. The email informs participants that stronger upper body muscles will help with "manual jobs around the home and in the garden" and reduce pain levels. The email asks participants, following completion of the exercise session, to repeat the session, and complete an arm raise challenge with instructions. | Education; Enablement. | Communications and marketing; Service provision. | 2.3 Self-monitoring of behaviour; 3.1 Social support; 4.1 Instructions on how to perform the behaviour; 5.1 Information about health consequences; 8.7 Graded tasks; 9.1 Credible source. |
| Video - Exercise session 5 - Upper body strength (33 minutes). | Exercise session on 'Upper body strength' with signposting to activity tracker and social media. | Education; Enablement; Training; Modelling. | Communications and marketing; Service provision. | 2.3 Self-monitoring of behaviour; 4.1 Instructions on how to perform the behaviour; 5.1 Information about health consequences; 5.2 Salience of consequences; 6.1 Demonstration of the behaviour; 8.7 Graded tasks; 9.1 Credible source; 13.2 Framing/reframing. |
| Email: Session 6 - Core strength. | Description of the session with signposting to the exercise video, the tracker booklet, activity hub, online community, and facebook group. The email informs participants that a stronger core can improve stability, increase movement and improve posture. The email asks participants, following completion of the exercise session, to repeat the session and to practice getting up from the floor. The email also specifically asks for participants to log their progress as they are now halfway through the programme. | Education; Enablement. | Communications and marketing; Service provision. | 2.3 Self-monitoring of behaviour; 3.1 Social support; 4.1 Instructions on how to perform the behaviour; 5.1 Information about health consequences; 9.1 Credible source. |
| Video - Exercise session 6 - Core strength (36 minutes). | Exercise session on 'Core strength' with signposting to activity tracker and social media and also back to the 'Getting started videos'. | Education; Enablement; Training; Modelling. | Communications and marketing; Service provision. | 2.3 Self-monitoring of behaviour; 4.1 Instructions on how to perform the behaviour; 5.1 Information about health consequences; 5.2 Salience of consequences; 5.6 Information about emotional consequences; 6.1 Demonstration of the behaviour; 8.7 Graded tasks; 9.1 Credible source; 13.2 Framing/reframing. |
| Email: Session 7 – Cardiovascular and respiratory fitness. | Description of the session with signposting to the exercise video, the tracker booklet, activity hub, online community, and facebook group. The email informs participants that cardiovascular fitness helps participants "live life to the full". The email asks participants, following completion of the exercise session, to repeat the 6-minute walking test, and to reflect on opportunities to get active outside of the exercise session. | Education; Enablement, Persuasion. | Communications and marketing; Service provision. | 2.3 Self-monitoring of behaviour; 3.1 Social support; 4.1 Instructions on how to perform the behaviour; 5.2 Salience of consequences; 9.1 Credible source. |
| Video - Exercise session 7 - Cardiovascular and respiratory fitness (34 minutes). | Exercise session on 'Cardiovascular and respiratory fitness' with signposting to activity tracker and social media. | Education; Enablement; Training; Modelling. | Communications and marketing; Service provision. | 2.3 Self-monitoring of behaviour; 4.1 Instructions on how to perform the behaviour; 5.1 Information about health consequences; 5.6 Information about emotional consequences; 6.1 Demonstration of the behaviour; 8.7 Graded tasks; 9.1 Credible source; 13.2 Framing/reframing. |
| Email: Session 8 - Balance and coordination. | Description of the session with signposting to the exercise video, the tracker booklet, activity hub, online community, and facebook group. The email informs participants of some of the health benefits of improved balance and coordination. The email asks participants, following completion of the exercise session, to complete a balance exercise and record the time in the activity tracker, inviting friends and family to take part. | Education; Enablement. | Communications and marketing; Service provision. | 2.3 Self-monitoring of behaviour; 3.1 Social support; 4.1 Instructions on how to perform the behaviour; 5.1 Information about health consequences; 9.1 Credible source. |
| Video - Exercise session 8 - Balance and coordination(33 minutes). | Exercise session on 'Balance and coordination' with signposting to activity tracker and social media. | Education; Enablement; Training; Modelling. | Communications and marketing; Service provision. | 2.3 Self-monitoring of behaviour; 4.1 Instructions on how to perform the behaviour; 5.1 Information about health consequences; 6.1 Demonstration of the behaviour; 8.6 Generalisation of target behaviour; 8.7 Graded tasks; 9.1 Credible source. |
| Email: Session 9 - Posture and stability. | Description of the session with signposting to the exercise video, the tracker booklet, activity hub, online community, and facebook group. The email informs participants that good posture can reduce pain. The email asks participants, following completion of the exercise session, to note tasks that affect posture, do a daily posture check, and schedule time to go for a walk. | Education; Enablement. | Communications and marketing; Service provision. | 2.3 Self-monitoring of behaviour; 3.1 Social support; 4.1 Instructions on how to perform the behaviour; 4.2 Information about antecedents; 5.1 Information about health consequences; 9.1 Credible source. |
| Video - Exercise session 9 - Posture and stability (41 minutes). | Exercise session on 'Posture and stability' with signposting to activity tracker and social media. | Education; Enablement; Training; Modelling. | Communications and marketing; Service provision. | 4.1 Instructions on how to perform the behaviour; 5.1 Information about health consequences; 5.2 Salience of consequences; 5.6 Information about emotional consequences; 6.1 Demonstration of the behaviour; 8.6 Generalisation of target behaviour; 8.7 Graded tasks; 9.1 Credible source; 13.2 Framing/reframing. |
| Email: Session 10 - Principals of training. | Description of the session with signposting to the exercise video, the tracker booklet, activity hub, online community, and facebook group. The email informs participants that good posture can reduce pain. The email asks participants, following completion of the exercise session, to complete the session again. The email asks participants following completion of the exercise session, to apply their knowledge of training principals to their own self-directed physical activity. | Education; Enablement. | Communications and marketing; Service provision. | 3.1 Social support; 4.1 Instructions on how to perform the behaviour; 9.1 Credible source. |
| Video - 10 - Principals of training (40 minutes). | Exercise session on 'Posture and stability' with signposting to activity tracker and social media. | Education; Enablement; Training; Modelling. | Communications and marketing; Service provision. | 4.1 Instructions on how to perform the behaviour; 5.2 Salience of consequences; 6.1 Demonstration of the behaviour; 8.6 Generalisation of target behaviour; 8.7 Graded tasks; 9.1 Credible source; 13.2 Framing/reframing. |
| Email: Session 11 - Flexibility. | Description of the session with signposting to the exercise video, the tracker booklet, activity hub, online community, and facebook group. The email informs participants that improved flexibility can make everyday tasks easier. The email asks participants, following completion of the exercise session, to complete the session again, and complete a sit-and-reach test daily. The email asks participants following completion of the exercise session, to apply their knowledge of training principals to their own self-directed physical activity. | Education; Enablement. | Communications and marketing; Service provision. | 3.1 Social support; 4.1 Instructions on how to perform the behaviour; 5.2 Salience of consequences; 9.1 Credible source. |
| Video - 11 – Flexibility (38 minutes). | Exercise session on 'Flexibility' with signposting to activity tracker and social media. | Education; Enablement; Training; Modelling. | Communications and marketing; Service provision. | 2.3 Self-monitoring of behaviour; 4.1 Instructions on how to perform the behaviour; 5.1 Information about health consequences; 6.1 Demonstration of the behaviour; 8.7 Graded tasks; 9.1 Credible source. |
| Email: Session 12 - Celebrating success. | Description of the session with signposting to the exercise video, the tracker booklet, activity hub, online community, and facebook group. The email asks participants to reflect on any changes from their notes in the activity tracker. | Education; Enablement. | Communications and marketing; Service provision. | 3.1 Social support; 4.1 Instructions on how to perform the behaviour; 9.1 Credible source. |
| Video - 12 - Celebrating success (46 minutes). | A full-body workout that covering all aspects of Leon’s programme. . | Education; Enablement; Training; Modelling. | Communications and marketing; Service provision. | 4.1 Instructions on how to perform the behaviour; 5.2 Salience of consequences; 6.1 Demonstration of the behaviour; 8.6 Generalisation of target behaviour; 8.7 Graded tasks; 9.1 Credible source; 13.2 Framing/reframing. |

a. BCT = Behaviour Change Technique; BCTTv1 = Behaviour Change Technique Taxonomy version 1.^1^ N.B: The Let’s Move with Leon activity hub, including the Let’s Move with Leon exercise videos and Activity Tracker, is available at <https://www.versusarthritis.org/about-arthritis/exercising-with-arthritis/lets-move-with-leon/>. The Let’s Move with Leon emails are available at <https://drive.google.com/drive/folders/1CWAAkS1HKoIN2m5FeE0Gm0CcTTuK0tOY?usp=sharing>.

Supplementary Table 2: Content of the Let’s Move with Leon Activity Tracker

| **Section** | **Brief description** | **Intervention function** | **Policy category** | **BCT and BCTTv1 code^a^** |
| --- | --- | --- | --- | --- |
| Getting started. | Detail provided of the general guidelines for physical activity. Information about the benefits of physical activity. Self-assessment of current physical activity levels (box to enter response). Assessment of barriers to physical activity (box to enter response) with self-identification of approaches to overcome the identified barriers (box to enter); identification of reward for overcoming barriers. Suggestion to 'Share your movements' on the facebook group. Use of a pain scale when active to ensure that pain does not exceed mild levels. Why is moving more important to you (box to enter)? A week calendar to select times to be active (tick by morning, afternoon or evening). | Education; Enablement. | Communications and marketing; Service provision, Guidelines. | 1.3 Goal setting (unspecified); 1.2 Problem solving; 2.3 Self-monitoring of behaviour; 3.1 Social support; 4.1 Instructions on how to perform the behaviour; 5.1 Information about health consequences; 9.1 Credible source; 10.7 Self-incentive. |
| Getting ready. | Information to consider in preparation for the first video such as wearing appropriate clothes, preparing a space for exercise, being well rested, taking medication and having a drink ready. | Education; Enablement. | Communications and marketing; Service provision. | 9.1 Credible source. |
| Week 1. | Explanation that it is normal to feel some soreness following exercise. Introduction of a whole body pain scale and a general pain scale with advice to stop if pain is rated as greater than 4/10 (tick box). Self-monitoring of exercises from the video completed in the week following the exercise session (box to enter response). Daily posture check chart to complete (tick box per day to enter response). Weekly task completion chart - 1 of 12 (tick box to enter response). | Enablement. | Service provision. | 2.3 Self-monitoring of behaviour; 2.6 Biofeedback; 9.1 Credible source. |
| Week 2. | Participants asked to reflect on their functional movements (box to enter response). Instruction to redo the exercise video, self-monitoring of the exercises completed (box to enter response). 6-minute walking task set with a space provided to record the results; encouraged to share on the facebook group. Self-monitoring of pain on a scale with advice to stop if pain is rated as greater than 4/10 . Weekly task completion chart - 2 of 12 (tick box to enter response). | Education; Enablement. | Communications and marketing; Service provision. | 2.3 Self-monitoring of behaviour; 2.6 Biofeedback; 3.1 Social support; 8.6 Generalisation of the behaviour; 9.1 Credible source. |
| Week 3. | Explanation that it is normal to feel some soreness following exercise. Importance of identifying own level and resting when necessary is highlighted. Daily posture check chart to complete (tick box per day to enter response). Self-reflection on strengths and weaknesses (box to enter response). Self-monitoring of pain on a scale with advice to stop if pain is rated as greater than 4/10 . Weekly task completion chart - 3 of 12 (tick box to enter response). | Education; Enablement. | Communications and marketing; Service provision. | 2.3 Self-monitoring of behaviour; 2.6 Biofeedback; 9.1 Credible source. |
| Week 4. | Explanation of the importance of lower-body strength. Instructed to repeat the exercise session and reflect on the repeated session (box to enter response). Instructed to complete a 1-minute sit-to-stand challenge on 3-days in the week (box provided to enter response). Self-monitoring of pain on a scale with advice to stop if pain is rated as greater than 4/10 . Weekly task completion chart - 4 of 12 (tick box to enter response). | Education; Enablement. | Communications and marketing; Service provision. | 2.3 Self-monitoring of behaviour; 2.6 Biofeedback; 8.6 Generalisation of the behaviour; 9.1 Credible source. |
| Week 5. | Explanation of the importance of upper-body strength. Instructed to repeat the exercise session and reflect on the repeated session (box to enter response). Instructed to complete a 1-minute arm-raise challenge on 3-days in the week (box provided to enter response). Self-monitoring of pain on a scale with advice to stop if pain is rated as greater than 4/10 . Weekly task completion chart - 5 of 12 (tick box to enter response). | Education; Enablement. | Communications and marketing; Service provision. | 2.3 Self-monitoring of behaviour; 2.6 Biofeedback; 8.6 Generalisation of the behaviour; 9.1 Credible source. |
| Week 6. | Explanation of the importance of core muscles for balance and stability. Instructions provided on how to get up and down from the floor. Instructed to repeat the exercise session and reflect on the repeated session (box to enter response), with a suggestion to also practice getting down and up from the floor with signposting to the video on the activity hub. Daily posture check chart to complete (tick box per day to enter response). Self-monitoring of pain on a scale with advice to stop if pain is rated as greater than 4/10 . Weekly task completion chart - 6 of 12 (tick box to enter response). | Education; Enablement. | Communications and marketing; Service provision. | 2.3 Self-monitoring of behaviour; 2.6 Biofeedback; 4.1 Instructions on how to perform the behaviour; 8.6 Generalisation of the behaviour; 9.1 Credible source. |
| Check-in | Comparison of physical activity from start of the programme to now (box to enter response); barrier review (box to enter response as to how barriers have changed); Goal review and resetting (box to enter response). Whole body pain scale and a general pain scale (tick box response). | Enablement. | Service provision. | 1.3 Goal setting (unspecified); 2.3 Self-monitoring of behaviour; 2.6 Biofeedback; 9.1 Credible source. |
| Week 7. | The aerobic physical activity guidelines provided. Instructed to repeat walking challenge from week 3 (box to enter response) with an increase in the challenge in some way (box to enter response). Self-monitoring of pain on a scale with advice to stop if pain is rated as greater than 4/10. Weekly task completion chart - 7 of 12 (tick box to enter response). | Enablement. | Service provision, Guidelines. | 2.3 Self-monitoring of behaviour; 2.6 Biofeedback; 9.1 Credible source. |
| Week 8. | Suggested to perform balance exercises on 2-days per week. Instruction to complete a balance exercise once a day measuring how long it can be held (box to enter response). Self-monitoring of pain on a scale with advice to stop if pain is rated as greater than 4/10 . Weekly task completion chart - 8 of 12 (tick box to enter response). | Education; Enablement. | Communications and marketing; Service provision. | 2.3 Self-monitoring of behaviour; 2.6 Biofeedback; 8.1 Behavioural practice/rehearsal; 9.1 Credible source. |
| Week 9. | Instructions on how to maintain a good posture. Participants asked to plan a time to go for a walk and record the time and how it went (box to enter response). Daily posture check chart to complete (tick box per day to enter response). Self-monitoring of pain on a scale with advice to stop if pain is rated as greater than 4/10 . Weekly task completion chart - 9 of 12 (tick box to enter response). | Education; Enablement. | Communications and marketing; Service provision. | 2.3 Self-monitoring of behaviour; 2.6 Biofeedback; 4.1 Instructions on how to perform the behaviour; 8.6 Generalisation of the behaviour; 9.1 Credible source. |
| Week 10. | Reminder of the physical activity training principles of frequency, intensity, time and type (FITT). Instructed to repeat the exercise session, recording the number of times (box to enter response). Encouraged to apply the FITT principles to an activity and record the adaptations made (box to enter response). Self-monitoring of pain on a scale with advice to stop if pain is rated as greater than 4/10. Weekly task completion chart - 10 of 12 (tick box to enter response). | Education; Enablement. | Communications and marketing; Service provision. | 2.3 Self-monitoring of behaviour; 2.6 Biofeedback; 4.1 Instructions on how to perform the behaviour; 9.1 Credible source. |
| Week 11. | Stretching can keep joints moving properly and reduce aches and pains. Participants asked to repeat the stretches from the exercise video two/three times a day, recording this (box to enter response). Instructions on how to perform a sit and reach test. Participants asked to complete the test, recording the reach once a day, up to seven days in the week (boxes provided to enter responses). Self-monitoring of pain on a scale with advice to stop if pain is rated as greater than 4/10. Weekly task completion chart - 11 of 12 (tick box to enter response). | Education; Enablement. | Communications and marketing; Service provision. | 2.3 Self-monitoring of behaviour; 2.6 Biofeedback; 5.1 Information about health consequences; 8.1 Behavioural practice/rehearsal; 9.1 Credible source. |
| Week 12. | Comparison of physical activity from start and middle of the programme (box to enter response); barrier review (box to enter response as to how barriers have changed); Goal review and setting of future goals (no timeframe given) (box to enter response). Whole body pain scale and a general pain scale (tick box response). Weekly task completion chart - 11 of 12 (tick box to enter response). | Enablement. | Service provision. | 1.3 Goal setting (unspecified); 2.3 Self-monitoring of behaviour; 2.6 Biofeedback; 9.1 Credible source; 10.7 self-incentive. |
| About Leon/  Keep in touch | Information about Leon and how to keep in touch with Versus Arthritis. | Enablement. | Service provision. | 9.1 Credible source. |

BCT = Behaviour Change Technique; BCTTv1 = Behaviour Change Technique Taxonomy version 1.^1^ N.B: The Let’s Move with Leon activity hub, including the Let’s Move with Leon exercise videos and Activity Tracker, is available at <https://www.versusarthritis.org/about-arthritis/exercising-with-arthritis/lets-move-with-leon/>. The Let’s Move with Leon emails are available at <https://drive.google.com/drive/folders/1CWAAkS1HKoIN2m5FeE0Gm0CcTTuK0tOY?usp=sharing>.

Supplementary Table 3: Data collection measures used at sign-up to Let’s Move with Leon

| **Outcome** | **Measure used** |
| --- | --- |
| Physical activity | Sport England Short Active Lives questionnaire^2^ |
| Quality of Life | Question 1(G1) from the World Health Organisation Quality of Life Brief Questionnaire^3^ |
| Self-efficacy | Single-item question from the Sport England Evaluation question bank^4^ |
| Confidence in maintaining lifestyle change | Question 13 from the Patient Activation Measure^5^ |
| Impact and ability to lessen impact of musculoskeletal condition on daily life | Two-item measure developed by Versus Arthritis |
| Belief that lifestyle changes will improve condition management | Single-item measure developed by Versus Arthritis |
| Knowledge of a healthy lifestyle | Single-item measure developed by Versus Arthritis |

Supplementary Table 4: How Let’s Move with Leon participants heard about the programme

| How heard | n | %^a^ |
| --- | --- | --- |
| An advert or article (facebook) | 9380 | 36.02% |
| Versus Arthritis social media | 8169 | 31.37% |
| Email from Versus Arthritis | 3039 | 11.67% |
| Versus Arthritis website | 2170 | 8.33% |
| Versus Arthritis publication (information booklet, leaflet or magazine Inspire/ Arthritis Today) | 1925 | 7.39% |
| Through a search engine (google etc.) | 982 | 3.77% |
| Another way | 830 | 3.19% |
| From a healthcare professional or NHS 111 | 724 | 2.78% |
| Word of mouth | 610 | 2.34% |
| Versus Arthritis sent something through the post | 183 | 0.70% |
| From a local Versus Arthritis volunteer / staff member | 107 | 0.41% |
| From another commercial organisation | 99 | 0.38% |
| From another charity | 74 | 0.28% |
| Multiple responses (included in the figures above) | 2852 | 10.95% |
| Can't remember | 376 | 1.44% |
| Data not provided | 225 |  |

^a: Percentages are calculated from the data available excluding missing and spoiled data from the total^

Supplementary Table 5: Gender of the Let’s Move with Leon cross-sectional survey participants by programme stage

| Gender | Female |  | Male |  | Other |  | Data not provided | Total |
| --- | --- | --- | --- | --- | --- | --- | --- | --- |
|  | n | % | n | % | n | % | n |  |
| Signed up but not started | 305 | 92.15% | 26 | 7.85% |  | 0.00% | 36 | 367 |
| Week 1-2 | 478 | 91.40% | 45 | 8.60% |  | 0.00% | 56 | 579 |
| Week 3-4 | 524 | 90.81% | 52 | 9.01% | 1 | 0.17% | 47 | 624 |
| Week 5-6 | 377 | 89.13% | 42 | 9.93% | 4 | 0.95% | 36 | 459 |
| Week 7-8 | 246 | 89.45% | 28 | 10.18% | 1 | 0.36% | 25 | 300 |
| Week 9-10 | 76 | 87.36% | 11 | 12.64% |  | 0.00% | 11 | 98 |
| Week 11-12 | 89 | 91.75% | 8 | 8.25% |  | 0.00% | 7 | 104 |
| End of programme | 189 | 91.30% | 16 | 7.73% | 2 | 0.97% | 14 | 221 |

^a: Percentages are calculated from the data available excluding missing and spoiled data from the total^

|  |  |  |  |  |  |  |  |  |  |
| --- | --- | --- | --- | --- | --- | --- | --- | --- | --- |
| Supplementary Table 6: Ethnicity of the Let’s Move with Leon cross-sectional survey participants by programme stage | | | | | | |  | |  |
| Ethnicity | White |  | All other ethnicities | | Data not provided | Total |  | |  |
|  | n | %^a^ | n | %^a^ | n |  |  | |  |
| Signed up but not started | 318 | 97.84% | 7 | 2.15% | 42 | 367 |  | |  |
| Week 1-2 | 500 | 97.47% | 13 | 2.53% | 66 | 579 |  | |  |
| Week 3-4 | 557 | 98.76% | 7 | 1.24% | 60 | 624 |  | |  |
| Week 5-6 | 406 | 98.07% | 8 | 1.97% | 45 | 459 |  | |  |
| Week 7-8 | 258 | 97.73% | 6 | 2.27% | 36 | 300 |  | |  |
| Week 9-10 | 83 | 98.80% | 1 | 1.19% | 14 | 98 |  | |  |
| Week 11-12 | 92 | 97.87% | 2 | 2.13% | 10 | 104 |  | |  |
| End of programme | 200 | 98.04% | 4 | 1.96% | 17 | 221 |  | |  |
| ^a: Percentages are calculated from the data available excluding missing and spoiled data from the total^ | | | | | | | |  |  |

| Supplementary Table 7: Age range of the Let’s Move with Leon cross-sectional survey participants by programme stage | | | | | | | | |
| --- | --- | --- | --- | --- | --- | --- | --- | --- |
| Age range | <35 | | 35-64 | | 65+ | | Data not provided | Total |
|  | n | %^a^ | n | %^a^ | n | %^a^ | n |  |
| Signed up but not started | 2 | 0.61% | 148 | 44.85% | 180 | 54.55% | 37 | 367 |
| Week 1-2 | 3 | 0.58% | 216 | 41.70% | 299 | 57.72% | 61 | 579 |
| Week 3-4 | 2 | 0.35% | 239 | 41.86% | 330 | 57.79% | 53 | 624 |
| Week 5-6 | 2 | 0.48% | 183 | 43.88% | 232 | 55.64% | 42 | 459 |
| Week 7-8 | 4 | 1.48% | 124 | 45.93% | 142 | 52.59% | 30 | 300 |
| Week 9-10 | 0 | 0.00% | 33 | 38.37% | 53 | 61.63% | 12 | 98 |
| Week 11-12 | 0 | 0.00% | 46 | 47.92% | 50 | 52.08% | 8 | 104 |
| End of programme | 3 | 1.46% | 78 | 38.05% | 124 | 60.49% | 16 | 221 |

^a: Percentages are calculated from the data available excluding missing and spoiled data from the total^

References used in the Supplementary Tables.

1. Michie S, Richardson M, Johnston M, et al. The behavior change technique taxonomy (v1) of 93 hierarchically clustered techniques: building an international consensus for the reporting of behavior change interventions. *Ann Behav Med* 2013; 46: 81–95.

2. Active Lives. *Sport England*, https://www.sportengland.org/know-your-audience/data/active-lives (accessed 23 November 2021).

3. WHOQOL - Measuring Quality of Life| The World Health Organization, https://www.who.int/tools/whoqol (accessed 23 November 2021).

4. Sport England Evaluation Framework - Sport England Evaluation Framework, https://evaluationframework.sportengland.org/ (accessed 23 November 2021).

5. Insignia Health (en-US), https://www.insigniahealth.com/products/pam-survey (accessed 23 November 2021).

Supplementary file 1 – Cross-sectional survey

1. I have the PHYSICAL opportunity to change my behaviour to improve my physical activity.

What is a PHYSICAL opportunity?

The environment provides the opportunity to engage in the activity concerned.

(e.g. sufficient time, the necessary materials, reminders)

Strongly disagree

Strongly agree

Please rate

0 1 2 3 4 5 6 7 8 9 10

☐ ☐ ☐ ☐ ☐ ☐ ☐ ☐  ☐ ☐

1. I have the SOCIAL opportunity to change my behaviour to improve my physical activity.

What is a SOCIAL opportunity?

Interpersonal influences, social cues and cultural norms provide the opportunity to engage in the activity concerned

(e.g., support from friends and family)

Strongly agree

Strongly disagree

Please rate

0 1 2 3 4 5 6 7 8 9 10

☐ ☐ ☐ ☐ ☐ ☐ ☐ ☐  ☐ ☐

1. I am motivated to change my behaviour to improve my physical activity.

What is motivation?

Conscious planning and evaluations (beliefs about what is good and bad)

(e.g. I have the desire to change, I feel the need to change)

Strongly agree

Strongly disagree

Please rate

0 1 2 3 4 5 6 7 8 9 10

☐ ☐ ☐ ☐ ☐ ☐ ☐ ☐  ☐ ☐

1. Changing my behaviour to improve my physical activity is something that I do automatically.

Automatic motivation involves doing something without thinking or having to consciously remember

(e.g. ‘is something I do before I realise I’m doing it’)

Strongly agree

Strongly disagree

Please rate

0 1 2 3 4 5 6 7 8 9 10

☐ ☐ ☐ ☐ ☐ ☐ ☐ ☐  ☐ ☐

1. I am PHYSICALLY able to change my behaviour to improve my physical activity.

What is a PHYSICAL capability?

Having the physical skill, strength or stamina to engage in the activity concerned.

(e.g. I have sufficient physical stamina, I can overcome disability, I have sufficient physical skills)

Strongly agree

Strongly disagree

Please rate

0 1 2 3 4 5 6 7 8 9 10

☐ ☐ ☐ ☐ ☐ ☐ ☐ ☐  ☐ ☐

1. I am PSYCHOLOGICALLY able to change my behaviour to improve my physical activity.

What is PSYCHOLOGICAL capability?

Knowledge and/or psychological skills, strength or stamina to engage in the necessary thought processes for the activity concerned.

(e.g. having the knowledge of why it is important, cognitive and interpersonal skills, having the ability to engage in appropriate memory, attention and decision making processes).

Strongly agree

Strongly disagree

Please rate

0 1 2 3 4 5 6 7 8 9 10

☐ ☐ ☐ ☐ ☐ ☐ ☐ ☐  ☐ ☐

Physical activity or exercise includes activities such as walking briskly, jogging, bicycling, swimming, or any other activity in which the exertion is at least as intense as these activities.

For activity to be regular, it must add up to a total of 150 minutes each week made up of bouts of 10 minutes or more; for example, you could take one 30-minute walk or take three 10-minute walks for a total of 30 minutes each day for five days.

1. I currently engage in regular physical activity.

Yes No

Questions taken from the following sources:

Keyworth C, Epton T, Goldthorpe J, et al. Acceptability, reliability, and validity of a brief measure of capabilities, opportunities, and motivations (‘COM-B’). *Br J Health Psychol* 2020; 25: 474–501.

Marcus, B. H., & Forsyth, L. H. (2009). Motivating people to be physically active. Leeds: Human Kinetics.
